# Attitudes towards working in retirement: a latent class analysis of older workers’ motives

**DOI:** 10.1007/s10433-020-00584-5

**Published:** 2020-10-09

**Authors:** Moritz Hess, Laura Naegele, Jana Mäcken

**Affiliations:** 1 Hochschule Niederrhein University of Applied Sciences, Mönchengladbach, Germany; 2 University of Vechta, Vechta, Germany; 3 University of Cologne, Cologne, Germany

**Keywords:** Working pensioners, Germany, Retirement, Transitions and old age potential (TOP), Working past retirement age, Social inequality

## Abstract

One of the fastest growing labour market groups is working pensioners, meaning those who work past the statutory retirement age whilst receiving a pension. Previous research has investigated the motives of this group and found very heterogeneous reasons for employment in retirement. However, little is known about the expectations and preferred work arrangements of older workers regarding a potential post-retirement employment. Using data from the German survey transitions and old age potential, we explore older workers’ motives, preferences and expectations towards working in retirement. Results show that about half of the respondents plan to work in addition to receiving a pension; however, the share is higher amongst men and those with higher levels of education. The motives for staying in post-retirement employment vary as well: using latent class analysis, we find four distinct patterns of motives that can be classified as (1) financially-driven, (2) status-driven, (3) contact and fun-driven, as well as (4) generativity-driven, underlining the complexity of retirement decisions. Furthermore, preferences regarding arrangements when combining work and retirement are very heterogeneous. Whilst highly educated men want to work as self-employed, women and those with lower qualifications want to stay in their old jobs. Only small differences were found regarding preferred hours (about 17) and days per week (2.24). In summary, the results show that the rapidly growing group of working pensioners and their preferences should be seen as characterised by differences by those responsible for creating these post-retirement employment opportunities.

## Introduction

In reaction to demographic ageing (Harper 2015), policymakers have introduced several pension and labour-market reforms aimed at delaying pension ages and extending working lives (Naumann 2014). They have increased official pension ages and closed early retirement options or tightened related eligibility criteria (De Tavernier and Roots 2015). In addition, they have tried to improve older workers’ employability through training programmes and measures such as life-long learning. It seems that these reforms are taking effect and pension ages, as well as employment rates of older workers, are rising all over Europe, although with large cross-country differences (Ebbinghaus and Hofäcker 2013). One of the fastest growing groups of older workers is those who work in addition to drawing a pension. As the number of pensioners working is rising, the boundaries between the social spheres of work and retirement are becoming increasingly blurred (Scherger 2015). The interpretation of this phenomenon varies widely: First, one could conclude that the rising number of post-retirement workers is a success story in the quest to fight age discrimination in the labour market. Second, others perceive these developments as a flexibilisation of the strict distinction between different phases of the institutionalised life-course (Naegele and Hess 2018; Kohli 1978), meaning that working beyond the pension age ‘challenges the fundamental meaning of old age, retirement, and old-age-related policies and, in a wider sense, also the institutionalized life course’ (Scherger 2015: 3). Following this argument, work in retirement can be conceptualised as a (new) stage in the transition to retirement that refers to the numerous resources older workers and retirees may still have to offer the job market (Wang and Shi 2014). Simultaneously, these kinds of employment (between complete retirement and complete working life) allow for greater flexibility, increase personal agency and enable older workers to shape their retirement according to their preferences (Froidevaux and Hirschi 2015). On the contrary, however, some researchers have argued this to be an exception to the social right to retire as a result of rising inequalities (Hess et al. 2016).

Though various determinants of post-retirement employment (demographic characteristics, health-, family-, or policy related variables) have been revealed by a growing body of research, fewer studies look at older workers’ motives and preferences in this regard. Preferences and motives are said to be time-bound, and it remains speculation whether people actually put them into practice. Nevertheless, they also reflect upon an individual’s employment history, contextual factors and subgroup affiliation. In this regard, researchers such as Mor-Barak (1995) and Baltes et al. (2012) highlight the importance of the `meaning of work´ when motives, preferences and expectations for (post-)retirement employment are being shaped. Research has shown that workers that experience work as meaningful are more productive, show higher job satisfaction, are more intrinsically motivated and have a higher organisational commitment (Steger et al. 2013). With focus on the group of older workers and retirees, research further has concluded that conducting meaningful work and finding meaning in life is of great importance to them (Shacklock and Brunetto 2011; Rosso et al. 2010) and that such meaning is shaped via an individual’s subjective interpretation of work experience and/or work-related interactions (Baumeister 1991; Wrzesniewski 2003). Combining Alderfer’s (1969) human needs theory with Erikson’s (1964) developmental theory, Mor-Barak (1995) derives four dimensions of work meaning (societal, personal, financial and generative) that have proven influential in post-retirement employment decisions. Amongst these, knowledge and experience transfer with younger generations (‘generativity’) has proven to be a strong driver for older people to stay employed until higher age (Fasbender et al. 2016). In contrast, research in a more sociological tradition proposes that people are more likely to deem work as meaningful if the social and cultural system around them ascribes value to the social sphere of work (Rosso et al. 2010). One could assume that within societies where cultural norms and pension regimes align with ‘active ageing norms’ (Hofäcker 2015) older workers are more likely to asign higher meaning in work that ultimately influences their choice to work in retirement.

Parallel to these findings, other studies state that employment opportunities and framework conditions being offered to older workers by employers and organisations shape preferences and affect employment behaviour in later life (Appelbaum et al. 2000; Anger et al. 2018). Furthermore, motives might differ between subgroups of older workers, resulting in differentiating preferences towards (future) working conditions and arrangements, and potential employment statuses. For example, it can be expected that workers with low pension benefits more often state financial motives as reasons for post-retirement employment. Therefore, it is advisable not to treat potential working pensioners as a homogenous group, but to differentiate them according to their socio-economic status.

Based on data derived from the German survey transitions and old age potential (TOP), the study at hand investigates older workers’ motives and preferences regarding the arrangement of a potential future post-retirement employment (work time, workplace and employment status). In addition, these motives and preferences are also further explored to see whether different socio-economic groups acknowledge potential inequalities in one’s ability to work after retirement. Thus, the following research questions were developed. (1) What determinants and motives for working beyond retirement can be identified? (2) Which preferences do future working pensioners state regarding working conditions and work arrangements? (3) Do the first two questions differ in relation to socio-economic status?

The contribution of the paper is threefold. By explicitly taking on a prospective perspective and looking at motives and preferences regarding post-retirement employment, a better understanding of the phenomenon and a chance for a more appropriate realisation of it in the future is gained. These findings might allow adjustments to work conditions regarding the special needs and preferences of (working) pensioners, either through creating more ‘agency’ on the side of the working pensioners or via addressing potential barriers and hindrances with employers and policymakers, ultimately preventing the potential clash of an individual’s expectations with actual future work environments. Second, through acknowledging differences in motives and preferences, it provides new insights into age-inclusive work environments, challenges ageist career paths and helps to prevent involuntary early labour market exits (Naegele and Hess 2018). In particular, the novel methodical approach of using latent class analysis (LCA) allows the identification of the main drivers of post-retirement employment. Third, although the rising risk of social inequalities in old age has been widely discussed in the context of retirement behaviour and/or timing (Hess 2018; Hofäcker and Naumann 2015), less is known about the risk of social inequalities in post-retirement employment. This paper aims to contribute to this debate by taking a closer look at different social groups of working pensioners.

## Setting the scene: the example of Germany

For several reasons, Germany is a very interesting case for analysing the preference of older workers for potentially staying employed beyond the pension age. First, the increase in absolute and relative numbers of working pensioners has been very steep, underlining the growing importance of this labour market group: Whereas the share of employed pensioners increased in Europe between 2000 and 2017 from 4.9 to 6.0%, it almost tripled in Germany from 2.7 to 7.0%, which also points towards an improvement in employment opportunities for older workers. Second, the policy shift towards extending working lives has been very drastic in Germany compared to other European countries (Hess et al. 2016). German policymakers increased the statutory retirement age from 65 to 67. In addition, early retirement via unemployment insurance and disability pensions was made financially much less attractive (Hofäcker and Naumann 2015). These measurements on the institutional level are accompanied by efforts to retain older workers at the company level. In particular, age-friendly human resources measures including, for example, health prevention programmes, age-inclusive training programmes, and ergonomically shaped workplaces are important tools that have been implemented (Frerichs et al. 2012). These efforts are reasonable considering the lack of skilled and qualified employees that is prominent in various sectors, such as high-technology, craft or—in the German case—especially in the sector of health and care (Naegele 2016). Utilising older workers and working pensioners is seen as one possibility to mitigate this problem (Naegele and Walker 2011). And as described, their labour force participation is increasing. However, these changes at the institutional and company level seem to affect groups of older workers differently. It seems that high-skilled workers benefit more from these changes, whilst low-skilled workers are struggling to meet the requirement of the new credo of late retirement (Naegele and Hess 2018). Concerns about the re-emergence of social inequalities in the late career phase and in the retirement transition have been voiced (Hess et al. 2016).

## Literature review and theoretical considerations

### Drivers of post-retirement employment

When looking at individual drivers behind post-retirement employment (Hofäcker and Naumann 2015; Scherger 2015), one can distinguish factors, determinants and antecedents and divide them into two main components: the *individual desire* and the *individual ability* to continue to work. Whereas the ability to continue to work refers to aspects of one’s individual health (Kim and Feldman 2000), qualifications and available labour market opportunities (Bäcker et al. 2017; Rump and Eilers 2017), the individual desire to work primarily discusses the financial aspects of retirement decisions. The overall financial situation, existing pension regimes, but also non-material work-related advantages (such as one’s individual enjoyment of work) seem to drive retirement behaviour (Hofäcker and Naumann 2015; Hess et al. 2016). At the individual level, several socio-demographic characteristics of working pensioners can be taken into consideration: Men work twice as often as women and higher qualification levels are associated with staying on beyond the statutory retirement age (Bäcker and Schmitz 2017). More recently, researchers have widened the understanding of drivers behind post-retirement employment by pointing towards more `soft factors´ such as social embeddedness and the intrinsic value of work. Based on the critique that the desire to work is solemnly based on expected income revenues, researchers have pointed out that work gives people a sense of purpose and identity, which ultimately affects their retirement preferences (De Tavernier et al. 2019; Radl 2013). Further, social embeddedness has proven an influence on retirement decisions. Family and care obligations, as well as being actively involved in volunteering responsibilities, moderate one’s desire to engage in post-retirement employment (Fasbender et al. 2016).

### Theoretical approaches to post-retirement employment

Different theoretical approaches can be used to explain post-retirement employment including, amongst others (1) the continuity-theory, (2) the work-role-attachment theory as well as the (3) cumulative disadvantage theory. According to Atchley’s continuity theory, ‘middle-aged and older adults attempt to preserve and maintain existing internal and external structures’ (Atchley 1989: 183), when making adaptive changes to their lives. They do so by using strategies which are tied to constructs and concepts they experienced in their past. Applied to the paper at hand, post-retirement employment can be interpreted as an opportunity to forego the disruptive life transition of retirement and, thus, maintain a familiar lifestyle with established daily routines (Bonsdorff et al. 2009; Kim and Feldmann 2000). Especially for individuals, who have been strongly committed to their work, retirement can have negative effects, e.g. inactivity or the loss of work-related networks. These individuals’ wish to seek some form of continuous engagement with their ‘work-life’ ultimately influences their decision to stay employed (Atchley 1989). In this regard, post-retirement employment presents itself as an extension of one’s current working life rather than a new career path with new tasks, new colleagues and a generally changed work environment (Naegele and Hess 2018). Based on the continuity theory, one would expect the main motives for potential post-retirement work to be the wish not to alter life too harshly and to remain in contact with the old workplace. With regard to the research question of employees’ preferences in their potential working conditions and arrangements during retirement, one would expect that they do not want to change these but would rather stay with the ones they know.

The work-role-attachment theory’s main argument is that the degree to which an individual is committed to their work affects the desire to remain a member of the workforce (Carter and Cook 1995; Adams et al. 2002). When applied to retirement transitions and the question of whether older workers wish to change career patterns, the three sub-dimensions of the work-role-attachment theory become of interest: job involvement, company identification and professional attachment (Carter and Cook 1995; Feldman 1994). If a worker has a high degree of job involvement, they tend to value their role as a holder within a particular job, whereas a worker who has a high degree of identification with a company merely wishes to stay a formal member of the same organisation. The third sub-dimension describes workers, who have a high degree of professional attachment and value their membership of a particular profession, which they do not necessarily have to carry out within the same organisation or position they held prior to retirement. Hence, based on the work-role-attachment theory, one would expect a large heterogeneity regarding the wishes to remain in employment and the arrangements for future working conditions.

According to the cumulative disadvantage theory, (dis)advantages earlier in the life-course entail (dis)advantages later which strengthen differences in socio-economic resources and status amongst social groups over time (Dannefer 2003; Crystal et al. 2016). In this regard, opportunities and motives for post-retirement employment differ depending on (dis)advantages experienced by the older worker over the life-course. Education is a main determinant in this respect. Low educated workers face poorer working conditions, ill health and lower income (Fisher et al. 2016). Thus, the motives and opportunities should differ depending on the older workers’ socio-economic status (Ferraro and Shippee 2009). Applying the cumulative disadvantage theory to the research question and keeping the changing German institutional context in mind, one would expect a variation of motives and preferences for planning to work in retirement by socio-economic status. Following concerns over social inequalities in retirement transitions, one would expect, on the one hand, that older workers from a lower socio-economic status more often state financial motives and prefer work settings which allow them to earn enough money. On the other hand, for older workers with a higher socio-economic status, non-financial motives should be more important.

## Data and methods

The analysis is based on data derived from the TOP study. TOP is a cross-sectional survey from 2013 in which older workers and pensioners were asked about their current/respectively past working context and past/future retirement transitions. Random sampling was carried out using the Gabler–Häder-Design (Sackreuther et al. 2016). Two different samples were used. First, we looked at all 1,868 respondents, who are currently working, and differentiated them according to those who wish to stay in employment during retirement and those who do not. Second, only the 744 respondents who are currently working and can imagine working after retirement were considered.

### Statistical analysis

The statistical analysis consisted of three steps. First, a logistic regression on the intention to work amongst all 1868 respondents was carried out. In the next step, an LCA for analysing the motives of the 744 respondents who can imagine working after retirement was estimated with the aim of identifying classes of motives. LCA is an explorative approach without making prior assumptions on the number and characteristics of classes. The result is a latent categorical variable that describes qualitative differences between classes, which are exclusive. Every respondent is assigned an individual probability of latent class membership. For instance, in a four-class solution, four probability values, which are one in sum, are generated for each individual. Every individual is then assigned to the class with the highest probability. Thus, the advantage of LCA compared to traditional cluster analysis is the explorative approach without prior assumptions. The LCA approach is based on detecting patterns based on probabilities and not on distance measures like traditional cluster analysis (Ellwardt et al 2016). Finally, linear and logistic regressions were used to investigate the preferences regarding the working conditions of a potential future post-retirement employment of the 744 respondents.

### Dependent variables

To answer the research questions about workers’ preferences regarding the working conditions of a potential future post-retirement employment, different dependent variables were used. The intention to work in addition to receiving a pension payment (yes/no) was considered first. If respondents answered yes, they were asked about their preferred working conditions including two variables on how many days and hours per week the older workers want to work. Both variables were treated as a metric, as they ranged from 1–7 days and 3–50 h. In addition, the respondents were asked if they wanted to work as self-employed (yes/no). The final dependent variable was if the older workers wanted to continue with their old job or preferred a new one (same/new one).

### Independent variables

The aim of the present study is to investigate motives behind a potential post-retirement employment. The three theories at hand propose different motives, which are crucial for working after retirement and range from the wish to stay in contact with others, the wish to pass on knowledge to the younger co-workers and the need to earn money. The first independent variable, thus, was the motives for a potential post-retirement employment. In the survey, the respondents were asked for the reasons why they planned to work after retirement. In total, nine reasons (continuing to earn money, stay mentally fit, having fun at work, contact with others, transfer of knowledge, recognition, feeling of being needed, having a daily routine and continuing education) were presented to the respondents and they were asked which of the reasons was important to them. Answers were coded on a four-point scale ranging from (Totally agree to Totally disagree). Answers were dichotomised using the median. In the next step, the answers were categorised using the LCA. The latent classes were included as dummies in the following analysis of the preferred working conditions.

Additionally, as the theory of cumulative (dis)advantage states that the motives likely vary between socioeconomic groups, the analysis was moderated by education. Education served as a proxy to distinguish between high and low socio-economic status groups as education summarises several interrelated characteristics that are important determinants of retirement decisions (Hofäcker and Naumann 2015). Education is measured using tertiles of years of education to group participants in low, medium and high levels of education. Low educated respondents had not more than 12 years of education, medium education ranged from 13 to 16 years and highly educated respondents had more than 16 years of education. To measure if class membership varies by education, interaction effects between class and low, medium and high education were included in the analysis.

### Control variables

Furthermore, previous research has shown that some variables are potential additional drivers of post-retirement work (e.g. Bäcker and Schmitz 2017; Fasbender et al. 2016; Hofäcker and Naumann 2015). Hence, analyses were controlled for respondents’ age, gender and marital status (having a partner/not having a partner), having caring obligations (yes/no) and self-rated health, which was measured on a four-point scale and dichotomised using the median. The current employment situation (civil-servant, self-employed or a blue-/white collar-worker) was also controlled.

## Results

### Descriptive results

Sample characteristics can be found in Table 1 and 2. They show that about half of the respondents plan to work in retirement. Of those who plan to work after retirement about 30% want to be self-employed and again 70% want to work in the same type of job. More highly educated want to be self-employed and the low educated want to do the same job in retirement as they did before.

**Table 1:** Descriptive overview of whole sample

*N* = 1868	Overall		
Variable	Mean	SD	Range
Intension to work	0.53	0.50	0–1
Men	0.50	0.50	0–1
Years of education	13.79	3.11	7–18
Age	58.34	2.87	54–70
Having a partner	0.81	0.39	0–1
Poor health	0.78	0.42	0–1
Caring obligations (yes)	0.30	0.46	0–1
Civil servant	0.13	0.33	0–1
Blue collar	0.11	0.32	0–1
White collar	0.60	0.49	0–1
Self-employed	0.16	0.32	0–1

**Table 2:** Descriptive overview of those who plan to work in retirement

*N* = 744	Overall			Low education		Medium education		High education	
Variable	Mean	SD	Range	Mean	SD	Mean	SD	Mean	SD
Same job (yes)	0.74	0.44	0–1	0.76	0.43	0.69	0.47	0.77	0.42
Future self-employment (yes)	0.30	0.46	0–1	0.17	0.38	0.38	0.49	0.39	0.49
Preferred working hours (weekly)	17.43	7.64	3–50	16.80	6.96	18.26	8.21	17.44	7.86
Preferred working days (weekly)	2.76	0.97	1–7	2.71	0.94	2.81	1.00	2.76	0.98
Men	0.51	0.50	0–1	0.45	0.50	0.53	0.50	0.58	0.50
Years of education	13.96	3.14	7–18	10.71	6.78	13.99	1.25	17.96	0.20
Age	58.54	2.94	54–65	58.41	2.90	58.43	3.10	58.80	2.83
Having a partner	0.78	0.41	0–1	0.78	0.41	0.76	0.43	0.78	0.41
Poor health	0.75	0.41	0–1	0.78	0.42	0.73	0.45	0.75	0.44
Caring obligations (yes)	0.30	0.45	0–1	0.29	0.46	0.31	0.47	0.30	0.46
Civil servant	0.10	0.30	0–1	0.03	0.16	0.08	0.27	0.20	0.41
Blue collar	0.09	0.28	0–1	0.18	0.38	0.06	0.23	0.01	0.11
White collar	0.60	0.49	0–1	0.68	0.48	0.60	0.49	0.53	0.50
Self-employed	0.21	0.41	0–1	0.14	0.35	0.27	0.44	0.25	0.43
Class 1: Financially-driven	0.41	0.49	0–1	0.47	0.50	0.38	0.49	0.38	0.49
Class 2: Status-driven	0.11	0.31	0–1	0.10	0.30	0.13	0.33	0.11	0.32
Class 3: Contact and fun-driven	0.32	0.47	0–1	0.30	0.46	0.31	0.47	0.35	0.48
Class 4: Generativity-driven	0.15	0.36	0–1	0.13	0.34	0.19	0.39	0.16	0.36

### Analysis of plans to work in retirement

The results of the first regression model (Table 3, left) show that more often men plan to work in retirement than women. Furthermore, older respondents, those not in a relationship, and the self-employed also have a higher probability of working in addition to receiving a pension. Civil servants and those with low education plan significantly less often to work in retirement. These findings are in line with previous research (Bäcker and Schmitz 2017; Bäcker et al. 2014).

**Table 3:** Regressions

	Intention to work (yes)	Same job (yes)	Future self-employment (yes)	Preferred working hours (weekly)	Preferred working days (weekly)
*Gender (ref: women)*					
Men	0.071^**^ (0.024)	− 0.011 (0.034)	0.060^*^ (0.030)	4.302^***^ (0.581)	0.286^***^ (0.076)
Age	0.010^*^ (0.004)	0.013^*^ (0.005)	− 0.006 (0.005)	0.236^*^ (0.092)	0.028^*^ (0.012)
*Education (ref: high)*					
Low education	− 0.012^***^ (0.036)	− 0.029 (0.038)	− 0.153^***^ (0.034)	− 0.205 (0.659)	0.014 (0.086)
Medium education	− 0.053 (0.035)	− 0.107^*^ (0.044)	0.004 (0.041)	0.872 (0.726)	0.054 (0.095)
*Relationship status (no partner)*					
Have a partner	− 0.110^***^ (0.029)	0.005 (0.039)	− 0.045 (0.034)	− 1.267 (0.659)	− 0.282^**^ (0.086)
*Health (ref: good)*					
Poor health	− 0.037 (0.027)	− 0.068 (0.039)	− 0.012 (0.032)	− 1.631^**^ (0.628)	− 0.175^*^ (0.082)
*Caring obligations (ref: no)*					
Yes	− 0.012 (0.025)	0.003 (0.034)	− 0.060 (0.031)	− 0.512 (0.583)	0.022 (0.076)
*Work status (ref: white collar)*					
Civil servant	− 0.169^***^ (0.035)	− 0.160^**^ (0.050)	− 0.021 (0.047)	− 1.807 (0.957)	− 0.010 (0.125)
Blue collar	− 0.064 (0.038)	− 0.080 (0.052)	− 0.019 (0.058)	0.156 (0.982)	0.051 (0.128)
Self-employed	0.160^***^ (0.034)	0.180^***^ (0.048)	0.400^***^ (0.021)	2.119^**^ (0.683)	0.303^***^ (0.089)
*Class (ref: class 1)*					
Class 2 Status-driven		− 0.067 (0.055)	0.085 (0.049)	− 0.386 (0.894)	− 0.022 (0.117)
Class 3 Contact-driven		0.007 (0.037)	− 0.046 (0.032)	− 2.684^***^ (0.637)	− 0.247^**^ (0.083)
Class 4 Knowledge gain-driven		− 0.055 (0.049)	− 0.032 (0.040)	− 3.061^***^ (0.806)	− 0.339^**^ (0.105)
*Interaction class × education*					
Class 2 × Low education		− 0.141 (0.082)	− 0.020 (0.066)	− 0.031 (1.325)	0.072 (0.173)
Class 2 × Medium education		− 0.010 (0.121)	0.021 (0.112)	− 2.513 (1.879)	− 0.322 (0.246)
Class 2 × High education		− 0.004 (0.091)	0.282^**^ (0.100)	0.742 (1.587)	0.076 (0.207)
Class 3 × Low education		− 0.029 (0.053)	− 0.056 (0.045)	− 1.770 (0.946)	− 0.165 (0.124)
Class 3 × Medium education		0.080 (0.082)	− 0.090 (0.073)	− 4.761^***^ (1.312)	− 0.433^*^ (0.171)
Class 3 × High education		0.003 (0.059)	− 0.004 (0.060)	− 2.370^*^ (1.101)	− 0.218 (0.144)
Class 4 × Low education		− 0.041 (0.073)	− 0.042 (0.058)	− 4.189^***^ (1.253)	− 0.488^**^ (0.164)
Class 4 × Medium education		0.014 (0.099)	− 0.046 (0.087)	− 4.586^**^ (1.516)	− 0.414^*^ (0.198)
Class 4 × High education		− 0.126 (0.085)	− 0.011 (0.077)	− 0.330 (1.417)	− 0.075 (0.185)
Observations	1868	744	744	744	744
*R*^2^	0.043	0.068	0.288	0.115	0.084

*p < 0.1; **p < 0.5; ***p < 0.01

### Analysis of motives for working in retirement

**Fig. 1:**
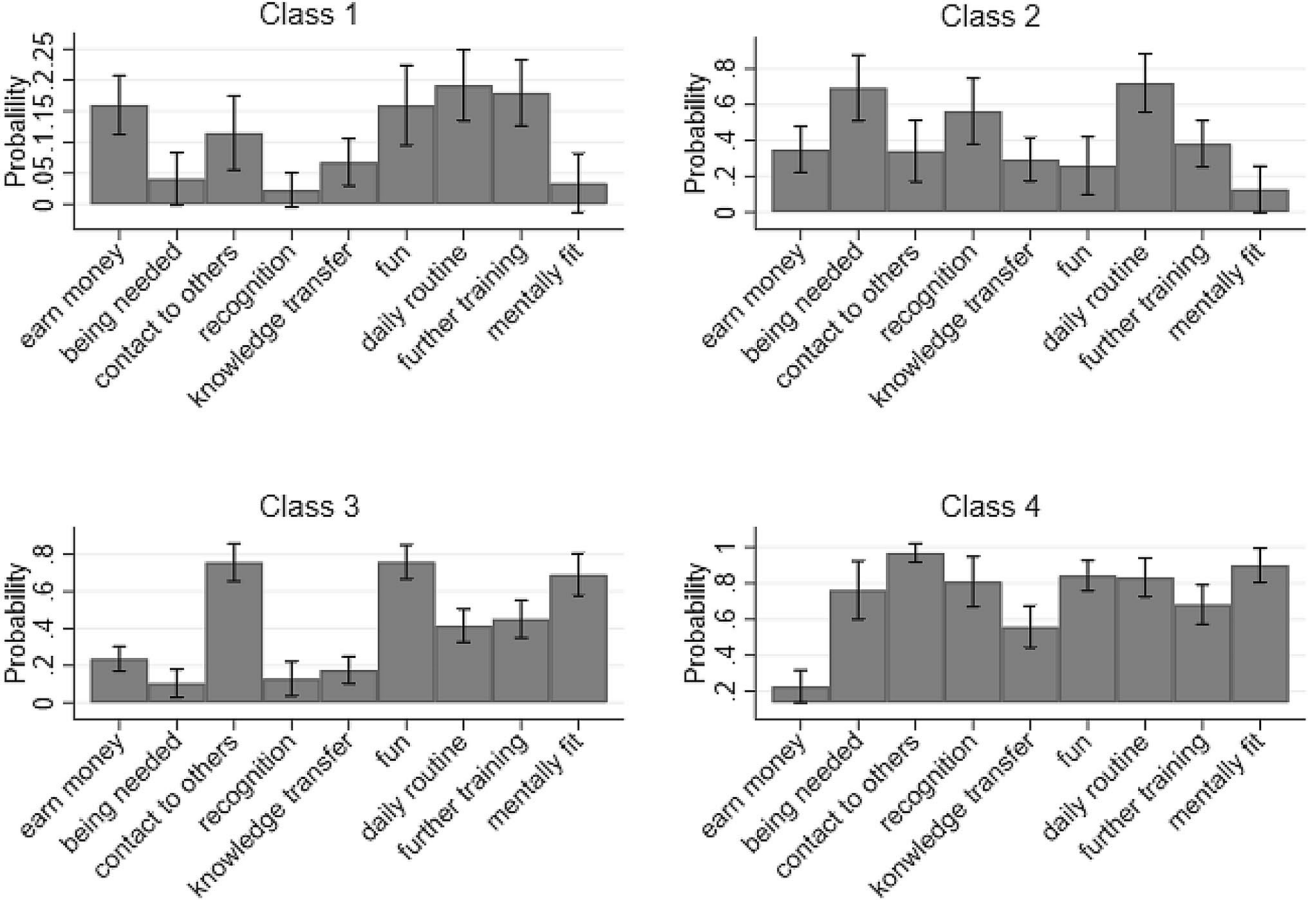
Distribution of classes based on LCA

Those who stated their intention to work in retirement were also asked for their reasons for doing so. Using the LCA, these reasons were then sorted into classes (Fig. 1). A four-class solution was considered the best according to the model fit. Class 1 comprises about 40% of the respondents and Class 3 about one third. Class 2 is made up of about 10% of the respondents and Class 4 of about 15% (for further information see: “Appendix 1”). The following class descriptions are identified based on an intra- and inter-comparison of the motives’ importance in each class:

Class 1: Financially-driven.

The first class is characterised by strong monetary reasons for staying in employment even in retirement. It seems that members of this class experience financial pressure as well as the wish for a daily routine, which is reflected in their motivation to stay in employment.

Class 2: Status-driven.

The wish for recognition and the feeling of being needed are comparably strong in the second class. To uphold one’s status as well as level of recognition seems to be the main driver for these class members to stay in employment when retired.

Class 3: Contact and fun-driven.

The third class mainly included those older workers who want to work in retirement because they enjoy work and want to stay in contact with their co-workers. Drivers of these class members’ willingness to stay employed in retirement, therefore, are more social than financial in nature.

Class 4: Generativity-driven.

To stay mentally fit and to participate in training and other work-related measures in the workplace drives the fourth class of members’ willingness to work in retirement. In addition, social contacts and the transfer of knowledge are, in comparison to the other classes, important drivers.

Bivariate chi^2^ tests for class membership and the control variables were conducted (for further information see: “Appendix 2”). Only gender and poor health status were significantly associated with class membership. Whereas in the financial and status-driven classes the gender ratio was almost balanced, more males than females were members in the contact and fun-driven as well as generativity-driven classes. Most respondents reported poor health in the contact-driven class followed by the knowledge-gain-driven class. Members in the status-driven class reported as the healthiest. The four classes were used in the next step as independent variables in regressions and which are depicted in the following.

### Analysis of the preferred working conditions in retirement

In the four regressions on the right side of Table 3, in which only those respondents were included who planned to work in retirement (*N* = 744), the preferred working conditions of work in retirement were explored. Men on average want to work more hours and days per week than women. The highly educated more often plan to work as self-employed. Amongst them are probably many consultants. The older the respondents are the more they want to do the same job and also work more, whilst those in poor health want to work less. The latter was to be expected, as poor health has been proven to be one of the main barriers to long working hours (Hofäcker and Naumann 2015). The association between age and longer working hours is also in line with previous studies about the preferred retirement age, which shows that older respondents prefer later retirement ages (Hess 2018). It seems that the closer one is to retirement the harder it is to leave work. The self-employed are a special group as they want to work more than all the others and also remain self-employed and in the same job. One explanation might be the high level of job and occupational identification amongst the self-employed (Binder and Coad 2016); they are also not included in the statutory pension system and hence might feel monetary pressure to delay retirement (Mäcken 2019). Different motives for working in retirement are clearly correlated with preferred working conditions. Those belonging to classes 3 and 4—the main motives being contact/fun and knowledge, respectively—state that they want to work significantly fewer hours and days than those in class 1—the main motives here being financial. The explanation might be that working more hours per week increases income, whilst it does not increase the utility of contacts and training opportunities as well as the possibility to pass on knowledge. This argument is supported by the results from the interaction effects. The effect of preferring less working time in class 3 is weaker for those with low education, meaning that even if their main motive is not financial it still plays a stronger role for them compared to those with medium and high education. Interestingly, the effect of belonging to class 4 is weaker for the highly educated. It seems that they want more opportunities to gain and pass on knowledge and, hence, plan to spend more time at the workplace. The final interaction effect found is that those belonging to class 2—the main motive here being status—and having a high education have a higher probability of wanting to work as self-employed. This is in line with previous findings that occupational identification and awareness of one’s status are important to older workers (Fasbender et al. 2016).

## Discussion

In summary, the results of the paper at hand show that about half of the respondents plan to work in addition to receiving a pension. The demographic variation in the plan to work in retirement is in line with previous findings from Germany (Bäcker and Schmitz 2017; Bäcker et al. 2014). The LCA found four different main motives for staying employed in retirement: financial, status, contact and fun as well as generativity. Differences regarding the preferred working conditions are rather small. Men do plan to work more days and hours per week than women, but no significant differences were found regarding favoured employer or job. Those with high education want to work more often as self-employed and those who are self-employed want to work more and stay in their current jobs. Those who intend to work in retirement due to monetary reasons also expect to work more hours and days per week. It seems plausible that this is due to the fact that more working hours increases one’s income (Hofäcker and Naumann 2015). And this effect is stronger for those with low education, indicating their need to delay retirement due to monetary pressure (Naegele and Hess 2018). The results show clearly that the group of working pensioners is very heterogeneous. They have different reasons for working in retirement and different preferences regarding their future working conditions. The first finding is reflected in qualitative research from Germany that also found a great variation in the reasons for working beyond retirement (Hokema and Scherger 2016).

Relating the results back to the three theories—continuity theory, work-role-attachment theory and the cumulative disadvantage theory—one comes to the conclusion that the ‘classical’ continuity theory (Atchley 1989) seems to apply mainly to self-employed in predicting older workers’ wishes regarding post-retirement work but not to others as these have been shown to be very heterogeneous. In addition, a substantial share of older workers does consider changing their type of work and also working as self-employed. The concept of opportunity can offer an explanation as the preference to change the type of work or being self-employed in retirement might stem from missing possibilities to work in the pre-retirement job (Fisher et al. 2016). This reorientation after retirement also suggests that pre and post-retirement work have to be seen as separate phases of the career and the life-course. The work-role-attachment theory, based on which one would expect the subsequent heterogeneity of the results (Adams et al. 2002), might offer explanations for large differences in older workers’ preferences regarding post-retirement employment and, thus, offers a suitable theoretical foundation when exploring older workers’ attitudes towards a potential post-retirement employment. The heterogeneity can be also interpreted from the perspective of the cumulative disadvantage theory (Dannefer 2003), as the results suggest that inequalities accumulate over the life-course and a large share of low educated older workers believes they have to work in retirement due to financial pressure. A combination of the work-role-attachment and the cumulative disadvantage theories might be a promising approach.

When interpreting the results, two main caveats must be acknowledged. First, the results only depict preferences and expectations of current workers regarding work in retirement and not the actual situation (Hofäcker 2015). This is of particular importance regarding the motives for potential employment in retirement. Second, preferences are only based on workers who plan to work after retirement. But, in particular, the low educated might not plan to work after retirement but be forced to do so, as they might have overestimated their actual pension level. Considering these two caveats the study still makes three contributions to the literature. It is one of few studies to investigate the motives and preferences regarding the potential post-retirement employment of older workers from a prospective perspective. In addition, it not only answers the questions of if and why older workers want to work in retirement, but also how they want to work. Second, it conducts a novel method of LCA analysis, which allows main classes of motives (and a more detailed exploration of those) to be identified behind post-retirement employment. And third, its focus on potential social inequalities sheds light on differences between socio-economic groups with regards to working in retirement.

Future research should further disentangle the groups of working pensioners, their motives and also the working conditions and arrangements they prefer. Particularly interesting is the group of self-employed which should be investigated separately, as they seem to have very distinct ideas and preferences regarding their post-retirement employment. In addition to the heterogeneity of future working pensioners, the topic of social inequality is also highly relevant to future research. This study shows that over one-third of respondents expect to work in retirement due to monetary reasons. Potential determinants and drivers of this inequality should be explored in more detail.

From a political and societal perspective, several implications can be drawn from the study. First, it can be interpreted positively that almost half of today’s older employees plan to work in retirement, as this means higher tax revenues and also helps to ease the lack of skilled labour that is currently a threat to the German economy (Naegele and Walker 2011). However, the study also supports recent warnings of social inequality in the retirement transitions found in previous research, in particular for Germany (Hofäcker and Naumann 2015; Hess et al. 2016), as a substantial share of workers expect to work in retirement for monetary reasons; and in particular for those who are in a vulnerable position anyway. Policymakers and other stakeholders should strive to mitigate these inequalities. Potential measures might include qualification and training, part-time retirement programmes and wage subsidies. In addition, employers also have to prepare for their ‘new employees’. Thus, it is imperative that companies aim for an age-inclusive as well as an age-appropriate work environment that allows for the flexibility needs of working pensioners. In addition, it is of utmost importance to abandon ageist career paths and ensure, by actively involving ageing pensioners in training and career development measurements, that those extended years in one’s working life are beneficial and fulfilling not only for the company but for the working pensioners themselves.

## Appendix 1

**Table:**

Number of classes	Fit statistics			
	Chi-square	AIC	BIC	df
1	1407.908	8302.022	8343.531	502
2	664.214	7578.328	7665.957	492
3	530.012	7464.127	7597.876	482
4	446.346	7400.461	7580.330	472
5	403.742	7377.857	7603.847	462

## Appendix 2

**Table:**

Class	1	2	3	4
*Gender*				
Female	59.5	50.6	38.8	39.1
Male	40.5	49.4	61.3	60.9
	Pearson *χ*^2^(3) = 28.0729 Pr = 0.000			
*Having partner*				
No	25.2	22.9	20.8	18.3
Yes	74.8	77.1	79.2	81.7
	Pearson *χ*^2^(3) = 2.8231 Pr = 0.420			
*Poor health*				
No	29.7	32.5	17.9	20.0
Yes	70.3	67.5	82.1	80.0
	Pearson *χ*^2^(3) = 14.2038 Pr = 0.003			
*Care obligations*				
No	67.7	63.9	74.6	69.6
Yes	32.4	36.1	25.4	30.4
	Pearson *χ*^2^(3) = 4.6680 Pr = 0.198			
*White collar*				
No	40.2	39.8	39.2	41.7
Yes	59.8	60.2	60.8	58.3
	Pearson *χ*^2^(3) = 0.2203 Pr = 0.974			
*Blue collar*				
No	89.9	92.8	91.3	92.2
Yes	10.1	7.2	8.8	7.8
	Pearson *χ*^2^(3) = 1.0068 Pr = 0.800			
*Civil servant*				
No	89.9	90.4	89.2	92.2
Yes	10.1	9.6	10.8	7.8
	Pearson *χ*^2^(3) = 0.8084 Pr = 0.847			
*Self-employed*				
No	80.1	77.1	80.4	73.9
Yes	19.9	22.9	19.6	26.1
	Pearson *χ*^2^(3) = 1.0068 Pr = 0.800			
